# Simulation of Organ Patterning on the Floral Meristem Using a Polar Auxin Transport Model

**DOI:** 10.1371/journal.pone.0028762

**Published:** 2012-01-23

**Authors:** Simon van Mourik, Kerstin Kaufmann, Aalt D. J. van Dijk, Gerco C. Angenent, Roeland M. H. Merks, Jaap Molenaar

**Affiliations:** 1 Biometris, Plant Sciences Group, Wageningen University and Research Center, Wageningen, The Netherlands; 2 Laboratory of Molecular Biology, Wageningen University and Research Center, Wageningen, The Netherlands; 3 Bioscience, Plant Research International, Wageningen University and Research Center, Wageningen, The Netherlands; 4 Centrum Wiskunde & Informatica, Amsterdam, The Netherlands; 5 Center for BioSystems Genomics, Wageningen, The Netherlands; 6 Netherlands Institute for Systems Biology, Amsterdam, The Netherlands; 7 Netherlands Consortium for Systems Biology, Amsterdam, The Netherlands; Lund University, Sweden

## Abstract

An intriguing phenomenon in plant development is the timing and positioning of lateral organ initiation, which is a fundamental aspect of plant architecture. Although important progress has been made in elucidating the role of auxin transport in the vegetative shoot to explain the phyllotaxis of leaf formation in a spiral fashion, a model study of the role of auxin transport in whorled organ patterning in the expanding floral meristem is not available yet. We present an initial simulation approach to study the mechanisms that are expected to play an important role. Starting point is a confocal imaging study of *Arabidopsis* floral meristems at consecutive time points during flower development. These images reveal auxin accumulation patterns at the positions of the organs, which strongly suggests that the role of auxin in the floral meristem is similar to the role it plays in the shoot apical meristem. This is the basis for a simulation study of auxin transport through a growing floral meristem, which may answer the question whether auxin transport can in itself be responsible for the typical whorled floral pattern. We combined a cellular growth model for the meristem with a polar auxin transport model. The model predicts that sepals are initiated by auxin maxima arising early during meristem outgrowth. These form a pre-pattern relative to which a series of smaller auxin maxima are positioned, which partially overlap with the anlagen of petals, stamens, and carpels. We adjusted the model parameters corresponding to properties of floral mutants and found that the model predictions agree with the observed mutant patterns. The predicted timing of the primordia outgrowth and the timing and positioning of the sepal primordia show remarkable similarities with a developing flower in nature.

## Introduction

In the past decade considerable progress has been made in understanding the mechanisms underlying the role of auxin and its transport mechanisms in the vegetative shoot apical meristem (SAM) and root apical meristem (RAM). Substantial experimental evidence suggests that auxin transport within the L1 layer of the SAM plays an essential role in organ initiation [1]. Laterally positioned leaves are initiated at auxin accumulation sites, established by active transport of auxin by PIN efflux carriers [2], [3]. After the switch from vegetative to generative development, floral meristems (FM) are initiated at the flanks of the central apical meristem, which from this stage on is referred to as inflorescence meristem (IM). Subsequently, the different floral organ primordia are initiated from the FM. Auxin plays an essential role in the initiation of floral organs and their spatial distribution [4]. Although the exact role and transport mechanisms of auxin in the FM have not been elucidated yet, small auxin drops that are artificially applied to the FM induce oversized flower primordia [2], and high auxin concentrations are observed in all young primordia [5]. This strongly suggests that the role of auxin in the FM is similar to the role it plays in the SAM, where local auxin maxima direct the initiation of organs.

Various hypotheses have been postulated in simulation studies on the details of auxin transport mechanics in general. In 2006, three papers by Jönsson, Smith, Barbier de Reuille, and their coworkers presented transport models in order to explain the phyllotactic pattern for leaf primordial initiation as found in the SAM [6]–[8]. These models are supported by experimental evidence, and are based on a concentration-based PIN polarization theory. This theory postulates that in each cell the PIN proteins in the membrane transport auxin into the neighboring cells, thereby favoring the neighbors with the highest auxin concentration. This up-the-gradient mechanism creates accumulation sites where organs are initiated. The models are based on early chemiosmotic concepts [9], [10]. Although precise parameter information is a subject of debate [11], these models demonstrate their capability of realistic pattern generation for stems and leaves, suggesting that the most important transport mechanisms have been included. Alternative to concentration-based models, flux-based models assume that PIN polarization is mainly driven by the direction of the flux, thereby amplifying existing fluxes. This model type is particularly successful in generating realistic venation patterns [9], [12]. A flux-based model was also proposed in meristem development to explain phyllotaxis [13]. A simulation study by Bayer et al. supports the hypothesis of intersecting flux-based and concentration-based mechanisms during formation of a midvein [14]. Wabnik and coworkers developed a model for PIN polarization that incorporates auxin signaling pathways in the apoplast [15].

The SAM and FM are both stem cell containing tissues, where PIN mediated auxin transport plays a crucial role in organ initiation. However, the organ initiation process in the FM differs from that in the SAM at some points. The main growth direction of the SAM is upward and each leaf primordium is placed at a certain distance and angle from the previous one, which creates a regular upward spiral pattern. In contrast, the FM expands radially during growth. Organs of the same type are not initiated consecutively, but more or less synchronously in the form of concentric whorls, and in a non-repetitive fashion. Smith et al. already showed that a polar auxin transport is able to generate repetitive whorled patterns [16]. In *Arabidopsis*, first the four sepal primordia appear in the outer whorl (first whorl), while later on the four petals appear in the second whorl positioned in an alternating fashion relative to the sepals, followed by six stamens and two carpels in the third and fourth whorls, respectively [17].

In this study we first present a confocal imaging study of the auxin accumulation patterns in a developing floral meristem. We observe that the auxin accumulations are predominantly restricted to the L1 layer, and that the auxin patterns resemble the floral organ pattern, both in spatial arrangement and in timing. Earlier, Reinhardt et al. [2] observed that PIN1 expression in young flower buds accumulated at positions and moments that coincided with the flower organ primordia and vascular strands. Also, small droplets of auxin that were applied to a *pin1-aux1* double mutant induced (oversized) flower primordia. From this it was concluded that there is a strong relationship between the development of an auxin concentration maximum and floral organ initiation. However, it should be realized that auxin accumulation apparently triggers organ initiation, but that, according to the so–called ABC model, the identity of the organ is determined by floral identity genes [18].

The second issue in this study is to investigate whether the typical patterning of auxin accumulations observed in the epidermis of the FM can be explained via simulations using an auxin transport model. Up to now, both concentration-based and flux-based auxin models have been successfully applied in predicting realistic patterns in the SAM. However, two putative auxin receptors, TIR1 and ABP1, respond to auxin concentration but are not likely to respond to auxin flux [19]. Therefore, we find concentration-based transport more plausible, and this is the reason why we adapted this model type here. This model class was first applied to phyllotaxis [6]–[8], and later extended to leaf venation patterning [19]. We made use of an integrated model and the software package VirtualLeaf [20] in which auxin transport and tissue growth are combined [19]. For the present purposes we extended this model with auxin induced cell growth, cell differentiation to a provascular cell type, and auxin transport into pro-vascular tissue.

To analyze the consequences of the proposed role and transport mechanisms of auxin, we conducted a simulation study of the growing floral meristem of *Arabidopsis*. The simulations show the development of auxin accumulation patterns on the growing FM as a function of growth rate and transport parameters. Assuming that a) auxin enters the young meristem from the stalk, and b) that for all cells the same transport rules apply, the predicted positioning and timing of the sepal primordia agree well with experimental observations. The subsequent primordia appear later than the sepals. To assess the predictive power of the model, we also investigated mutant type flowers and adjusted the model parameters according to the mutant properties. Also for mutants the similarity between model predictions and observed mutant patterns suggests that auxin transport suffices for floral patterning.

## Results

### Experimental observations

**Figure 1:**
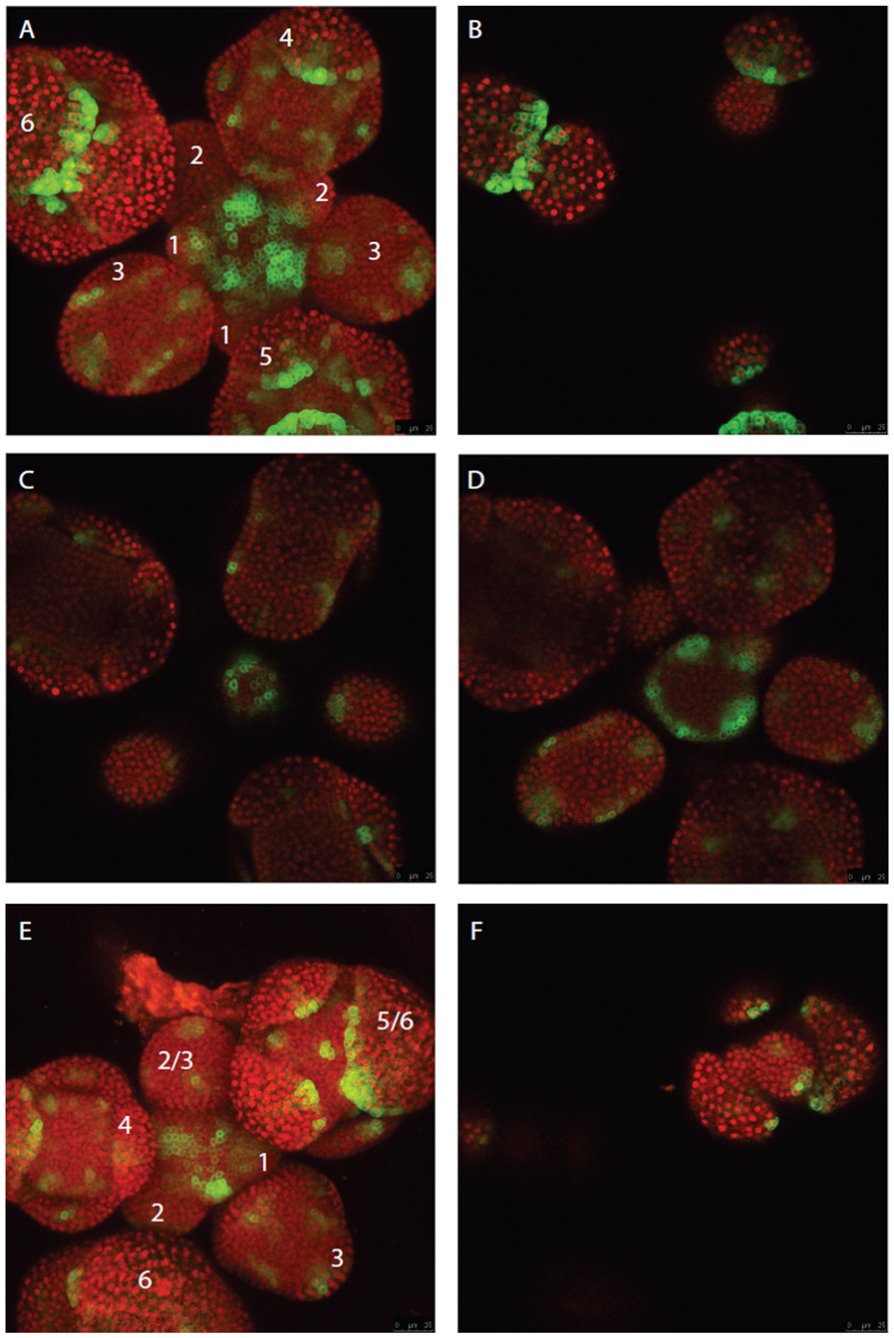
Auxin distribution at early stages of flower development revealed by DR5rev::GFP expression. A,E: 3D projection of ER-localized GFP signal (green) and constitutively expressed, nuclear localized TagRFP signal (red) in young inflorescence tissues. Numbers indicate floral stages according to [17]. B–D) individual Z sections of 3D projection shown in (A), descending in Z location. F) individual Z section of 3D projection shown in (E).

For indirect monitoring of auxin distribution in the meristematic cells, we used transgenic lines expressing a reporter construct containing Green Fluorescence Protein (GFP) fused to the auxin inducible promoter from the DR5 gene [21]. In line with being a major site of auxin accumulation, the inflorescence meristem showed strong, highly patterned DR5 signal (Figure 1 A, E). The signal was mostly confined to the L1 layer, and peaks of signal coincided with floral pre-primordia in a spiral phyllotactic pattern (Figure 1 C, D, and the supplemental movie). During these earliest stages of floral initiation, DR5 signal becomes detectable in sub-epidermal layers, where it becomes gradually restricted to cells giving rise to the pro-vasculature (Figure 1 D). In stage 1 of flower development (floral stages according to [17]), DR5 signal is generally strongly reduced compared to the pre-primordial stages (Figure 1 A, E). The signal is mostly confined to the adaxial side of the floral primordium, in L1 daughter cells of the DR5 expressing cells in the pre-primordium. A weak DR5 signal in other L1 cells in the floral primordium indicates reorganization of auxin accumulation/response. In floral stage 3, sepal primordia arise, with the two abaxial and adaxial sepals arising first, and the lateral primordia growing out afterwards. Sepal initiation coincides with the formation of DR5 peaks in the same temporal order (Figure 1 A, C, D). At later stages of sepal development, DR5 expression becomes mostly confined to L1 cells in tips of the growing sepal organs, and to internal cells likely giving rise to pro-vascular strands (Figure 1 B). In floral buds of stage 4, four auxin accumulation sites can be recognized, which are alternating with the position of the sepals and represent the four petal primordia (Figure 1E). At the transition of stage 4 to 5, DR5 signal peaks, comprising a small set of cells in the more central part of the FM and marking the initiation of the 4 medial stamen primordia (Figure 1 A, C, D) and DR5 signal is present in the L1 cells and in cell layers below, indicating again the formation of pro-vasculature. In deeper layers of the meristem the DR5 signal indicates common pro-vasculature strands of petals and stamens (Figure 1 D). In the central part of the FM at stage 5 to 6, weak DR5 signal throughout the L1 and the formation of more strong peaks indicates reorganization of auxin accumulation/response ultimately triggering initiation of the carpel primordial around stage 6 (Figure 1 A, F). This stage marks the cessation of meristematic growth and the transition towards floral organ growth and differentiation. Figure 1A reveals that in stages 1 and 2 auxin accumulates predominantly in the half of the meristem that is attached to the stalk. A continuous descending view through the z-stacks in Figure 1 is presented as Movie S1 on the PLoS website, and cross sections of Figure 1 E are presented as Text S1 and Figure S1.

### Auxin transport model

The up-the-gradient auxin transport model we used is based on the theory that auxin is pumped through the cell walls by PIN efflux carriers located at the cell membrane. Therefore, the efflux rate of auxin depends on the number of PIN molecules at the membrane. The PIN molecules cycle between the membrane and an intracellular compartment called the endosome. Auxin inhibits endocytosis of PIN's in neighboring cells, thereby directing auxin fluxes towards cells with a higher auxin concentration than their neighbors [22]. As counterpart of this active transport we define passive transport as transport that is not mediated by PIN molecules, and can be modeled as cell-to-cell diffusion. Passive transport tends to level out concentration differences between cells, whereas active transport amplifies them. The ratio of passive and active transport typically determines the distance between two accumulation sites [6]. Increasing the contribution of active transport turns out to lead to a decrease in distance. Simulation studies [6] show that a combination of both transport mechanisms in the L1 layer of the SAM explains the phyllotactic patterning of organs that are initiated during the vegetative stage of development. We assume that the growth rate of a cell increases with its auxin concentration [23]. We also assume that, after a cell has reached a threshold auxin concentration, it differentiates into an organ primordial cell and auxin is pumped from the L1 layer into the underlying pro-vascular tissue, that starts to act as an auxin sink [2]. This assumption is similar to Smith's et al., who constrained the auxin under a maximum in organ primordia [7] to mimic auxin flow into deeper tissue layers. Barbier de Reuille et al. introduced auxin sinks at fixed sites where vascular strands are observed via PIN1 immunolocalization images [8].

The resulting auxin transport and cell growth model is spatially homogeneous, i.e., the same rules apply to all cells. Based on the observation that during stages 1 and 2 auxin seems to be predominantly active in the meristem hemisphere attached to the stalk, we make the simplifying assumption that during stage 1 auxin enters the meristem from the stalk. Furthermore, we restrict the simulations to the part of the meristem surface where the primordia develop. This part is curved upward and has, from a top view, more or less a circular shape that expands in time due to dividing and expanding of the meristem cells. We apply the simulations to a circular shaped planar approximation of the meristem. The consequences of this approximation are discussed under ‘simulation results’. Auxin entering the meristem surface from beneath enters this circular shape via the cells at the boundary. The equations for auxin transport and PIN localization are
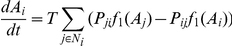


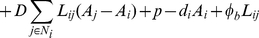
(1)

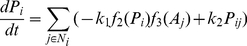
(2)

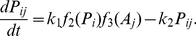
(3)The first equation describes the auxin concentration per cell, the second and the third describe the cycling of the PIN molecules between the endosome and the cell walls. Auxin concentration 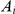 is the concentration of auxin in cell 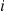, which depends on 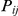, the amount of PIN molecules in cell 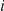 at the wall facing neighbor 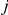. The PIN molecules cycle between wall and endosome, and 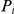 is the amount of PIN in the endosome. We define 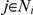 as the set of cells 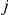 adjacent to cell 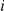. Further, 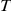 and 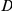 are the active and passive transport coefficients, 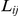 the cell wall surface, and 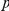 is a constant local auxin production. The term 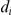 accounts for both decay and depletion after a primordium has initiated: if 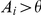, then 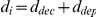 with 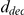 and 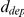 decay and depletion, respectively. Parameters 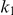 and 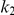 characterize the PIN cycling between endosome and cell membrane, and 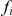 are Michaelis-Menten saturation functions. 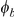 Represents the auxin influx at the perimeter of the simulation domain, i.e., this term reflects auxin fluxes through the L1 layer towards the tip of the floral meristem. This influx is active only for a small period of time in the first stage of development. The cell surface increases with
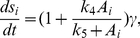
(4)with 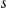 the surface area, 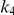 and 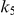 the auxin-dependent growth rate and the half maximum value respectively, and 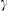 the growth rate constant. The growth of the cell depends on the resulting turgor pressure, the strain of the cell walls, and the pressure from surrounding cells. The cell growth is determined by solving the minimal energy problem
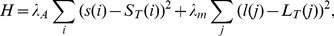
(5)with 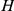 the generalized potential energy, 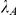 describes turgor pressure resistance, 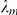 is a spring constant, 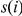 and 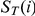 the actual cell area and predefined (fixed) resting area respectively, and 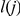 and 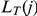 the actual wall length and predefined resting length, respectively. Indices 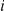 and 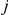 sum over all cells and polygon edges, respectively. After 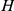 is minimized, auxin transport and cell expansion and -division are computed. Cell division occurs once the cell area has doubled since the last division, creating a round “callus” of meristem tissue [20]. Equation (5) is minimized using a Monte Carlo based algorithm that generates a set of random deformations of the current cell shapes. The algorithm is described in more detail in [20]. After a cell has undergone a division, the daughter cells inherit the auxin level of the parent. A more detailed description of the model equations are provided in Text S2; Installation and compilation of the VirtualLeaf framework [20] is described in Text S6. Installation files are given as File S1.

### Simulation results

**Figure 2:**
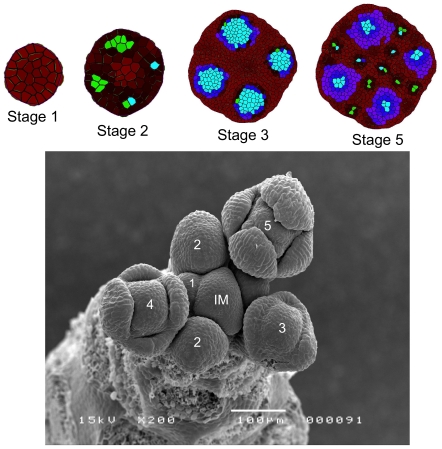
Top: Simulated auxin accumulations on a growing meristem. Green indicates auxin levels above 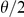, red indicates the amount of PIN in a cell endosome, and differentiated cells are marked in blue. More brightness indicates higher auxin concentration. Bottom: scanning electron microscopy image of an *Arabidopsis* shoot with floral meristems.

We used parameter values that resemble the values available in the literature. The ratio between diffusion and active transport, 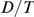, is close to the one used in [19], and the ratio of the PIN cycling parameters 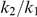 resembles the value that was reported in [6]. The period of auxin inflow, and cell growth rate are calibrated to our experimental observations. Further details are provided in Text S3 and Table S1. When the unknown parameter values are tuned on *Arabidopsis* wild type, our approach predicts auxin accumulation peaks that resemble observed organ patterns. Figure 2 shows the simulated auxin accumulation patterning on a developing meristem from stage 1 to stage 5. Illustration of the complete simulation is presented as Movie S2 on the PLoS website. First, four accumulation sites appear in the outer ring. Note that this patterning occurs automatically, since the auxin influx is uniform along the equator of the meristem. In reality, the sepal organs appear pair-wise instead of simultaneously. By using nonsymmetric inflow we could not generate pair-wise patterning. When the auxin concentration in a cell surpasses a threshold value (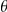), the cell is assumed to differentiate into an organ primordial cell. The positions of the four accumulation sites that form first suggest that they must be associated with sepal tissue. The sepal primordia grow substantially until cell growth and division in the FM have stopped. From our experimental observations we estimate that meristem growth slows down substantially when the number of cells is about 700 (see Text S3). Then, additional, smaller concentration maxima appear. The position of these smaller concentration maxima is directed by the position of the initial, larger auxin maxima.

We associate the maxima in between the sepal primordia with petal primordia, and those in the meristem center with the reproductive organs. In *Arabidopsis*, the numbers of sepals, petals, stamens, and carpels are 4-4-6-2, respectively. Since equation (5) is solved with a Monte-Carlo algorithm, i.e., stochastic, the resulting topology varies per run. Comparing the positions of the auxin maxima in the model with the positions of organs on a real *Arabidopsis* meristem, we conclude that on average our simulations yielded the correct 4 sepal positions. For the other primordia more variation is observed with as tendency that there are slightly more than 4 auxin maxima on petal positions and less than 6+2 on reproductive organ positions. This last outcome is at least partly due to the 2D approximation of the meristem, since the planar disc used in the simulation has less space near the center than the real meristem. We expect that this is also the reason why the presumed petal primordia are located more to the outside than in reality. As for the timing we find good agreement with our experimental observation: the maxima associated with petals and reproductive organs appear three stages later than the maxima associated with the sepals (see Figure 2).

#### Robustness and sensitivity

**Figure 3:**
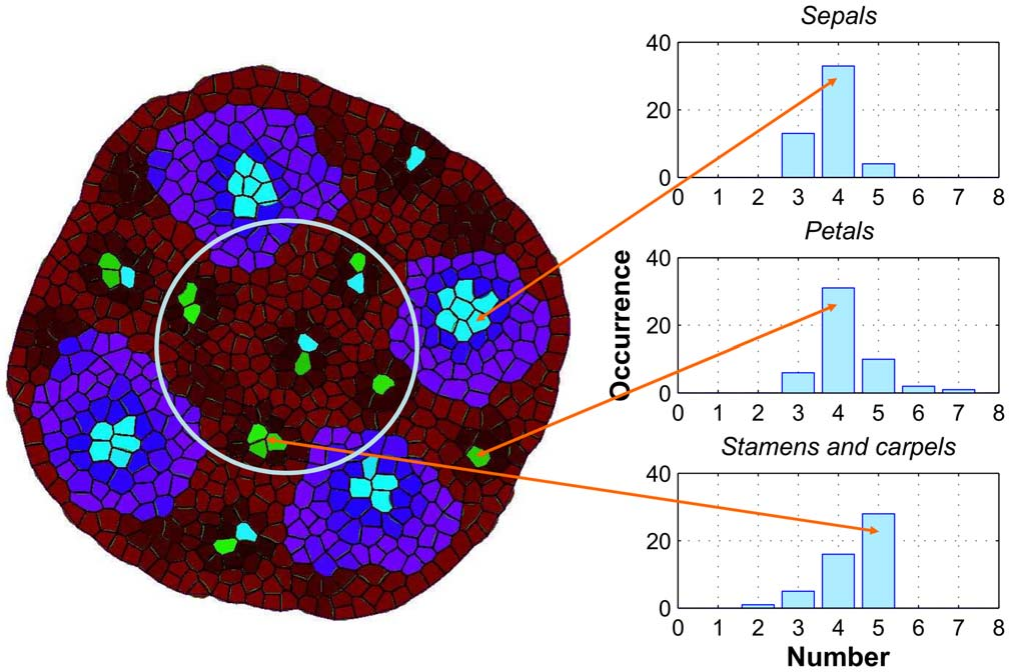
Bar graph of the emerging organ numbers for 50 simulations. The arrows indicate the organs on the simulated meristem. The circle separates the areas of the outer two whorls and inner two whorls. The color coding is the same as in Figure 2.

**Figure 4:**
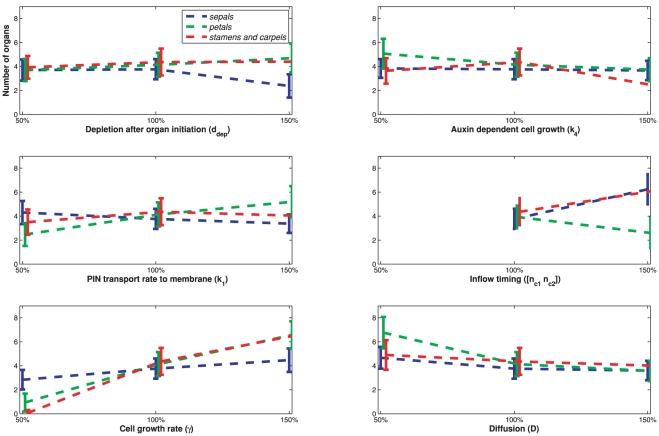
Predicted organ numbers after variation of 6 parameters (interpolated with dotted lines). The bars denote standard deviations of the predictions (solid lines).

Because we make use of a stochastic growth and cell division algorithm [20], all simulations have a random component. The robustness of the pattern formation is assessed by performing a batch simulation of 50 runs. In each run the number of meristem cells increases from 4 to 700. Two auxin peaks are associated with different organs if the cells are separated by at least one cell with less than the threshold concentrations, or alternatively if they come into existence at different times. The organ identities associated with the auxin maxima are determined based on their locations. We projected a circle onto the meristem to separate the maxima representing organs in the outer two whorls (sepals and petals) from those representing the reproductive organs (stamens and carpels). Within the outer two whorls, initiation sites are associated with sepals if they emerge before stage 2, and the others are defined as petals. Sometimes two separate auxin maxima fuse after some time, and are then considered as one presumed organ. Figure 3 shows a bar graph of the organ types and numbers. We find a variation of 30% in sepal and petal number, and 50% in the reproductive organs. The pattern 4–4–5 (sepals–petals–stamens and carpels) occurred most often (16 times), followed by 4–5–5 and 4–4–4 (both 5 times). Also in nature the numbers of flower organs in wild type *Arabidopsis* vary, but to a much lesser extent: the number of petals has about 1% variation and the number of stamens about 7% variation measured over 100 flower samples [24]. The sensitivity of the model is assessed by varying six key model parameters: diffusion (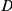), cell expansion rate (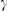), auxin inflow timing at the equator (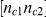), transport of PIN from endosome to cell membrane (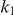), auxin dependent growth (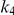), and depletion rate after organ initiation (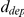). Figure 4 shows the effect on the organ numbers. The parameters are set to 50, 100 and 150% of their nominal values (the values that are found for the wildtype simulations), and for each value the average prediction of 30 simulations is displayed, together with their standard deviations.

The timing of the auxin influx via the boundary turns out to be a crucial factor. The auxin flows in as long as the number of meristem cells is in the range [50, 100]. Then, four sepals are formed. If this range is halved ([25, 50]), one or two large undefined initiation sites form at positions that seem neither related to sepals or other any other organ type. The auxin dependent growth 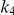 is small relative to the growth rate constant 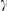, and hence cell growth is almost uniform. Variation of 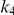 does not influence the patterning very much, and hence it does not seem to be an essential factor. Different parameters affect the numbers of organs in different ways. This indicates that in principle different parameter sets could fit to the wild type organ pattern. The values of the threshold value 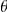 and depletion rate after organ initiation 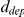 are optimized, so their influence on pattern predictions deserves some attention. We observed that the auxin pattern is robust against extreme variations of 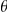. We found the best fit for values of 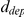 in the regime for which the pattern is robust against variations. Even though in this regime the depletion rate is already 10 times higher than that of ordinary decay, it takes even higher values of 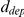 to influence the pattern considerably (Text S4 and Figure S2).

### Simulation of mutants

Some mutant plants have known defects in either auxin biosynthesis or transport machinery, and their molecular deficiencies as well as their phenotypes have been reported. So these mutants are ideal to test the predictive power of the model. As a validation study, we adjust the model parameters according to these deficiencies, and compare the model predictions with the reported phenotypes. Based on our experimental finding that organ initiation is associated with an elevated auxin level, we assume in this section that cells that are differentiating (blue) and cells with stable, highly elevated auxin levels (bright green) are directly related to organ initiation. Organ identities are based on locations of the maxima in the same way as for wildtype. Figures of the patterns are presented in Text S5 and Figures S3, S4, and S5. Simulations are presented as Movies S3, S4 and S5 on the PLoS website.

#### Weak mutants

**Figure 5:**
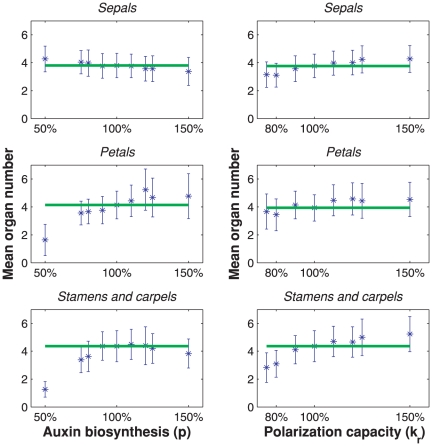
Bar graph of organ numbers as function of auxin biosynthesis 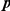 (left), and polarization capacity 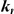 (right). The bars denote standard deviation of the predictions (asterisks), and the green lines denote the organ numbers for the nominal parameter set.

First, we consider two mutants *yucca (yuc)* and *pinoid (pid)* that show only moderate deviations in organ numbers. These mutants can be simulated by adjusting the parameters only moderately. Originally, the *yuc1yuc4* mutant severely lacked auxin biosynthesis and was a strong mutant that showed almost no floral organs (on average 0.7 organs per whorl [4]). However, in another experiment the *yuc1yuc4* mutant was partly rescued by a 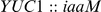 construct. The results suggest a still suboptimal auxin production. The sepal-petal-stamen-carpel number was 4 - 2.4 - 5 - 1 on average (10 plants). We mimick the effect of changing auxin biosynthesis in our model predictions by shifting parameter 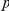 from 50% to 150% of its standard value (and the inflow parameter 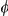 accordingly); see Figure 5 (left). Every prediction is based on the average outcome of 30 simulations. We find that, in agreement with the mutant phenotype, a small decrease in auxin biosynthesis leads to a decrease in petal-, stamen- and carpel numbers, whereas the sepal number stays fairly constant. In all predictions the standard deviation is considerable, but clear trends are visible.

The PINOID gene is associated with PIN protein polarization. Overexpression of the gene causes altered sensitivity to or transport of auxin [25]. The *pinoid (pid)* mutants even show a switch in PIN protein polarization [26], [27]. We simulated a weak mutant by decreasing polarization parameter (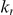), assuming that for a weak mutant the polarization capacity decreases. Figure 5 (right) shows that a moderate decrease in polarization capacity predicts a trend in organ numbers that agree with observations for the weak mutant *pid-8* [28]. This mutant hardly affects sepal- and petal number, but the average stamen number is on average reduced to 4.

#### Strong mutants

**Figure 6:**
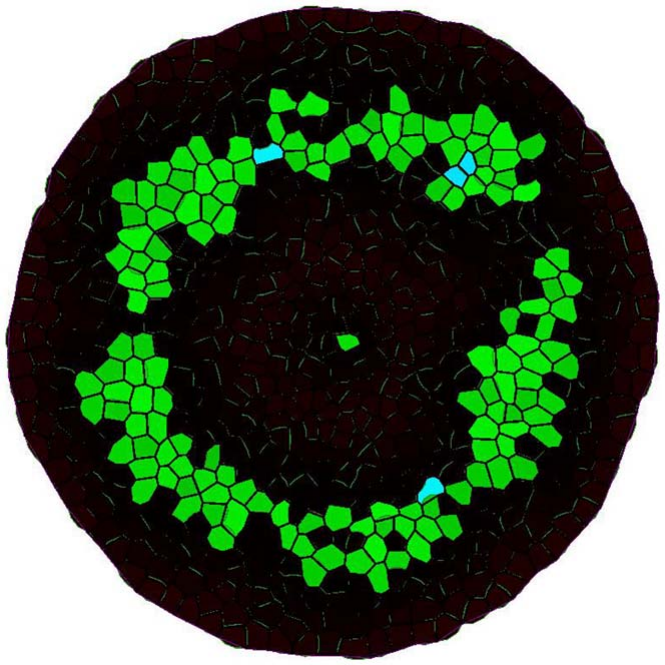
Simulation result for the strong *pin* mutant in stage 5. The color coding is the same as in Figure 2.

The *pin-formed-5 (pin-5)* and *pin-formed-3 (pin-3)* mutants have defects in the PIN1 protein, and show a dramatic change in organ numbers. *Pin-5* mutants have a typical organ number of 6-5-1 (the number of carpels was not reported), with a large variation. *Pin-3* flowers have a typical organ number of 6-10-0, also with a large variation [28]. To check whether our simulations show the same tendencies, we decrease the fixed amount of PIN1 per cell down to 20% of its original value. This huge adjustment has considerable effect. The simulations no longer yield clearly separated auxin maxima, but more or less concentric rings of cells with elevated auxin levels show up (Figure 6). Inside this ring little or no initiation sites are formed, which is in agreement with the absence of whorl 3 and 4 organs in *pin-3* mutants. From Figure 6 we cannot deduce organ numbers. Although we cannot compare predicted and observed organ numbers in this case, we may conclude that the model predicts the correct tendency, i.e., a dramatic increase in sepal and petal numbers.

## Discussion

Patterns of DR5 reporter expressions support the hypothesis that in *Arabidopsis* auxin accumulations induce organ primordia initiation on the floral meristem. For wild type *Arabidopsis*, the present transport model predicts realistic organ patterning on a determinate growing meristem. The model is validated by weak and strong mutants, thereby giving an explanation of how the mechanisms that are altered in these mutants cause different patterning. Furthermore, using a parameter sensitivity analysis the model predicts the influence of various physical alterations on organ numbers. The simulation model correctly predicts positions of sepals. A key mechanism observed in our model, is that the sepals direct the positions of the smaller auxin maxima associated with petals, stamens, and carpels. Initial organs act as templates for the positioning of subsequent organs. Thus, auxin transport along the L1 layer in the growing floral meristem may be responsible for positioning of the floral organs. Crucially, we need to assume that auxin flows into the floral meristem after it has formed around 50 cells. A similar assumption was made in the phyllotaxis models proposed by Jönsson et al. [6] and Smith et al. [7] who assumed a central zone at the tip of the shoot apical meristem where no auxin is produced. Here we hypothesize this inhibition of auxin production is lifted at the (terminal) floral meristem before the floral meristem reaches its final size. Thus, the terminal differentiation into a floral meristem, associated with prolonged auxin production, may suffice to induce a switch from phyllotactic patterns at the SAM, to the whorl like auxin maxima in the floral meristem. Similarly, by inducing a stricter dependence of PIN1 production on auxin concentration, the same upstream auxin pumping mechanism can drive leaf venation patterning [19]. Thus, depending on context and parameter settings controlled by upstream genes, the auxin upstream pumping mechanism or related auxin transport polarization mechanisms [15], [29] could be responsible for organ positioning during phyllotaxis [6], [7] and floral development, and venation patterning in the leaf [19].

The reliability of the model was further tested by predicting the phenotypes of *pin, pid* and *yucca* mutants. These mutants all have deficiencies in the auxin transport process and auxin biosynthesis, which could be mimicked by adjustment of the corresponding parameters in the model. Sensitivity analysis showed that the simulated patterns are quite robust against parameter variations. The variation in floral organ numbers in nature is substantially smaller than what is predicted by the simulations, which could be caused by an unknown stabilizing mechanism, or by missing details on a cellular level. The variation in predicted organ numbers hardly depends on the specific parameter choice. It should be noted that the mutants used here often have considerably high organ variations. In other words, suboptimal parameter conditions can decrease robustness of the biological system. For example, the percentages of variation in the sepal-petal-stamen number in the rescued *yuc1yuc4* plants are 0%-50%-60% [4], in the *pid-8* plants 30%-30%-60%, in *pid-1* 60%-70%-70%, and in *pid-2* 60%-60%-70% [28]. Except for the sepal variation in *yuc1yuc4* plants, these percentages are in the range of the predicted variations (30%-30%-50% for wild type and similar for mutants). This aspect deserves further investigation. Although from this study the most essential pattern mechanism seems to be polar transport, various refinements and extensions are possible. One refinement would involve taking into account the curved shape of the meristem instead of performing the simulations on its planar projection. This aspect seems in particular to be essential for a more precise prediction of the number of reproductive organs, and investigation of the role of auxin transport from the stalk to the young flower bud. Another refinement regards the assumption that the transport rules are uniform over the meristem and constant in time. However, time- and organ-specific auxin production rates were observed in young floral organs [5]. A partial explanation could be found in additional interactions. There are several downstream targets of auxin that play crucial roles in floral organ development. Examples are AINTEGUMENTA and the AINTEGUMENTA-LIKE family, which are downstream transcription factors of auxin that have different expression patterns. In petunia, auxin may be a downstream target of the FLOOZY protein, which is first expressed in the center of the floral meristem, and later on the flanks of the initiating petal and stamen primordia and at several sites in maturing stamens and carpels [30]. Also, evidence has been found for a link between the floral identity genes APETALA1 and SEPALLATA3, that are expressed at specific times and locations within the meristem, and auxin-related transcription factor expression [31], [32]. Including these factors into the model could help explaining the role and expression levels of auxin for different stages in different organ tissues. Finally, quantitative parameter information could allow us to test different possible biological hypotheses rigorously, for example the role of auxin transport mechanisms within the apoplast, or, together with a 3D model, the role of mechanisms behind vascular strand formation. Altogether, this study provides novel insights into the parameters that are important for floral organ initiation and patterning and forms the basis for experimental validation of their precise role.

## Materials and Methods

The localization of the auxin marker DR5rev::GFP [21] and constitutively expressed NLS-TagRFP were determined in inflorescence meristems and floral meristems up to floral stage 6 (stages according to [17]). The inflorescence material was dissected and embedded in 0.1% agar. Confocal laser scanning microscopy of the living plant tissue was performed with a Leica SPE DM5500 upright microscope with a ACS APO 40×/1.15 lens, using the LAS AF 1.8.2 software (Leica). Scanning Electron Microscopy (SEM) was performed as described previously [33]. For the auxin and PIN dynamics in equation (1) a fifth order Runge-Kutta algorithm was used [20].

## Supporting Information

Figure S1 **Auxin distribution at early stages of flower development revealed by DR5rev::GFP expression.** The cross sections in the x-y, y-z and x-z plane are presented in each subfigure. The white dotted lines denote how the planes are located relative to each other, and where they intersect.
(TIF)

Figure S2
**Top:** 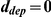 **(left) and** 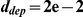 **(right). Bottom:** 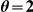 **(left) and** 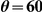 **(right).**
(TIF)

Figure S3 **Resulting patterns of the wildtype simulations.** Green denotes elevated auxin level, blue corresponds to differentiated cell. Brighter green or blue indicates higher auxin levels.
(TIF)

Figure S4 **Resulting patterns of the** ***yuc*** **mutant simulations.**
(TIF)

Figure S5 **Resulting patterns of the** ***pid-8*** **mutant simulations.**
(TIF)

File S1 **Supporting information software.** Auxinsimon.cpp, Auxinsimon.h, Auxinsimon.pro, and Basis-floral_ortime.xml used for model simulation.
(ZIP

Movie S1 **Descending view of the DR5::GFP expression in young flower buds.**
(WMV)

Movie S2 **Simulation of wildtype.**
(WMV)

Movie S3 **Simulation of rescued** ***yuc1yuc4*** **.**
(WMV)

Movie S4 **Simulation of** ***pid-8*** **.**
(WMV)

Movie S5 **Simulation of** ***pin-5*** **.**
(WMV)

Table S1 **Nominal parameter values used in the simulations.**
(TIF)

Text S1 **Experimental observations.**
(TEX)

Text S2 **Auxin transport model.**
(TEX)

Text S3 **Model calibration.**
(TEX)

Text S4 **Robustness.**
(TEX)

Text S5 **Prediction of mutant phenotypes.**
(TEX)

Text S6 **Software compilation.**
(TEX)
