# Supplementary figures and images for: Simulation of Organ Patterning on the Floral Meristem Using a Polar Auxin Transport Model
Source: PLoS One. 2012 Jan 23;7(1):e28762. doi: 10.1371/journal.pone.0028762 (PMC3264561; doi:10.1371/journal.pone.0028762)

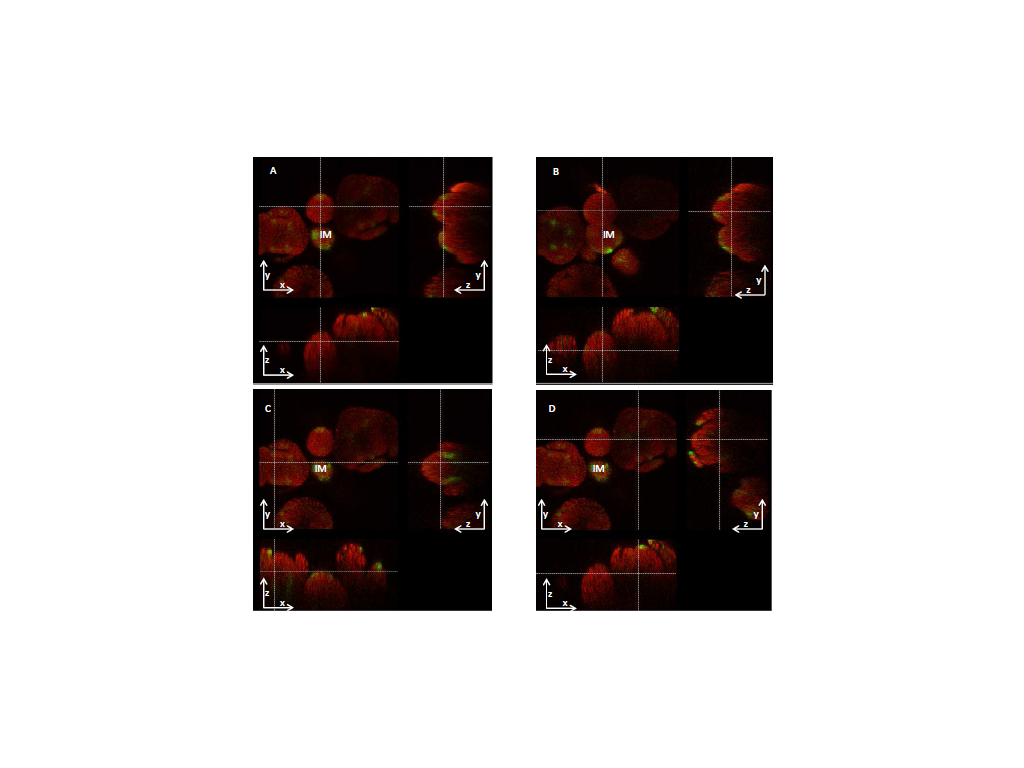

Supplement: Figure S1 — Auxin distribution at early stages of flower development revealed by DR5rev::GFP expression. The cross sections in the x-y, y-z and x-z plane are presented in each subfigure. The white dotted lines denote how the planes are located relative to each other, and where they intersect. (TIF) [file pone.0028762.s001.tif]

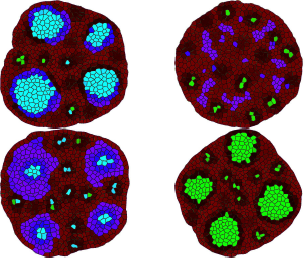

Supplement: Figure S2 — Top: (left) and (right). Bottom: (left) and (right). (TIF) [file pone.0028762.s002.tif]

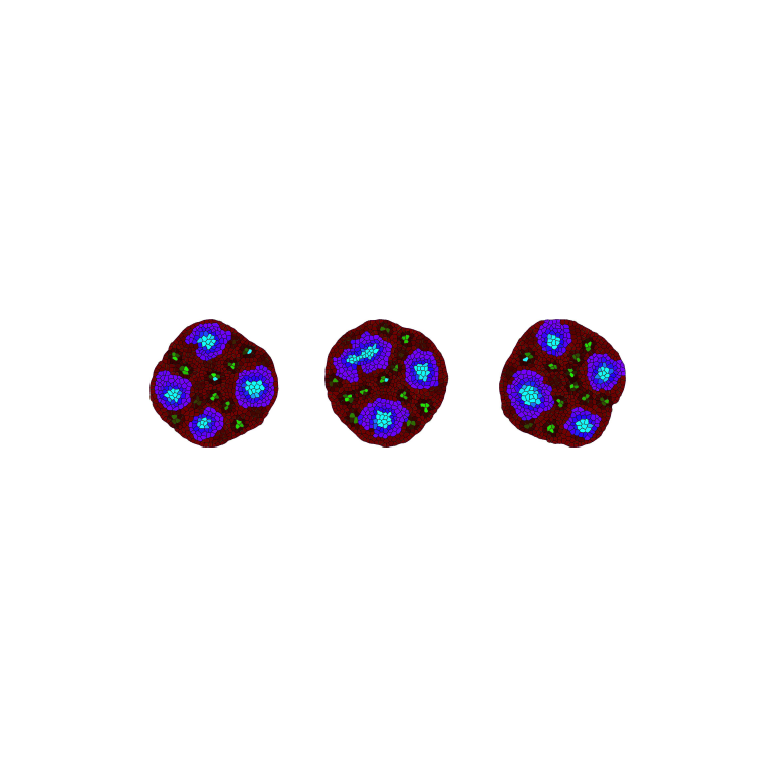

Supplement: Figure S3 — Resulting patterns of the wildtype simulations. Green denotes elevated auxin level, blue corresponds to differentiated cell. Brighter green or blue indicates higher auxin levels. (TIF) [file pone.0028762.s003.tif]

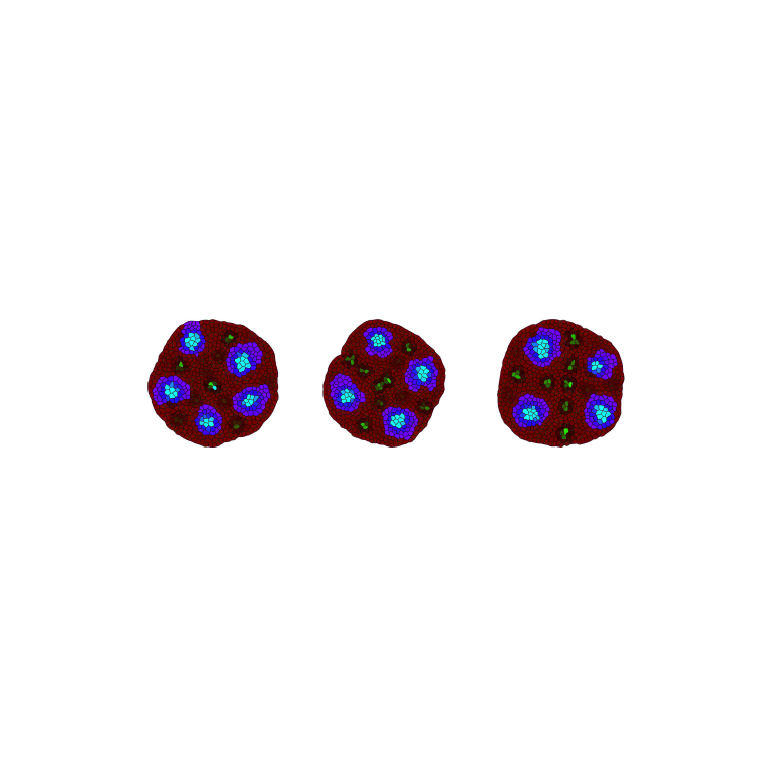

Supplement: Figure S4 — Resulting patterns of the yuc mutant simulations. (TIF) [file pone.0028762.s004.tif]

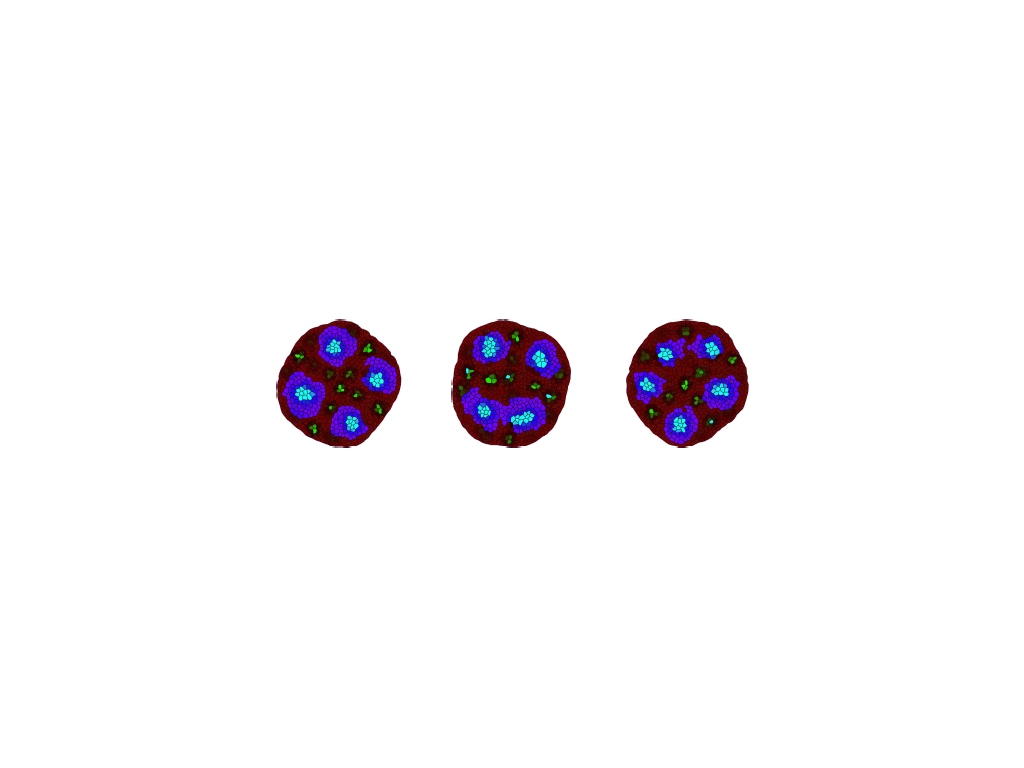

Supplement: Figure S5 — Resulting patterns of the pid-8 mutant simulations. (TIF) [file pone.0028762.s005.tif]

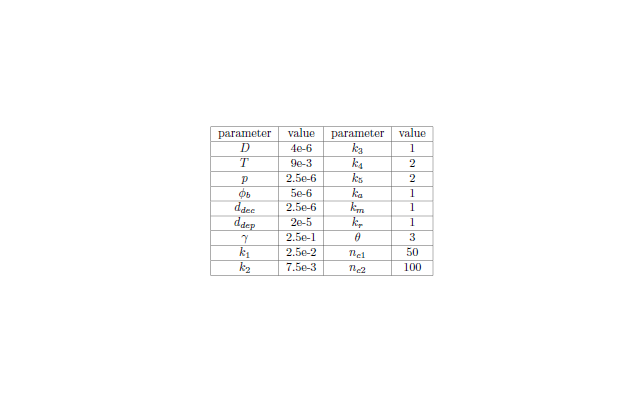

Supplement: Table S1 — Nominal parameter values used in the simulations. (TIF) [file pone.0028762.s012.tif]
